# Design and Evaluation of Novel Peptides Targeting Protein Disulfide Isomerase as Antiplatelet Agents

**DOI:** 10.3390/molecules31193516

**Published:** 2026-10-02

**Authors:** Francisca Rodríguez-Cabello, Diego Méndez, Eugenia Ulzurrun, Abigail E. Teitgen, Felipe Lagos, Fanny Guzmán, Andrés Trostchansky, Basilio Carrasco, Nuria E. Campillo, Iván Palomo, Eduardo Fuentes

**Affiliations:** 1 Thrombosis and Healthy Aging Research Center, Department of Clinical Biochemistry and Immunohematology, Faculty of Health Sciences, University of Talca, Talca 3460000, Chile; francisca.rodriguez@utalca.cl (F.R.-C.); diego.mendez@utalca.cl (D.M.); felipe.lagos@utalca.cl (F.L.); 2 Margarita Salas Biological Research Centre, Spanish National Research Council (CSIC), Ramiro de Maeztu 9, 28040 Madrid, Spain; mariaeugenia.ulzurrun@cib.csic.es (E.U.); abigail.teitgen@csic.es (A.E.T.); nuria.campillo@csic.es (N.E.C.); 3 Instituto de Ciencia de Materiales de Madrid, Spanish National Research Council (CSIC), C. Sor Juana Inés de la Cruz 3, 28049 Madrid, Spain; 4 Núcleo Biotecnología Curauma, Pontificia Universidad Católica de Valparaíso, Valparaíso 2373223, Chile; fanny.guzman@pucv.cl; 5 Departamento de Bioquímica and Centro de Investigaciones Biomédicas (CEINBIO), Facultad de Medicina, Universidad de la República, Montevideo 11800, Uruguay; 6 Centro de Estudios en Alimentos Procesados (CEAP), Av. Lircay s/n, Talca 3480094, Chile; bcarrasco@ceap.cl

**Keywords:** protein disulfide isomerase, targeted peptides, rational design, thrombosis, molecular dynamics, covalent inhibitor, drug discovery

## Abstract

Extracellular protein disulfide isomerase (PDI) is a critical mediator of thrombus formation and a promising target for novel antithrombotic therapies aiming to overcome the bleeding risks associated with current antiplatelet agents. This study aimed to design and evaluate novel, targeted peptide-based PDI inhibitors with a safe physiological profile. We utilized a bioinformatics-driven rational design approach, based on the evolutionary conservation of the PDI active site, to generate a library of 34 targeted peptides. The inhibitory mechanism and biological efficacy of the lead candidate, peptide p23, were evaluated using 500 ns molecular dynamics (MD) simulations, flow cytometry, and in vitro microfluidic thrombosis models under arterial shear stress. Based on bioinformatic analyses, p23 is predicted to interact with the PDI catalytic cleft and potentially form a highly stable inhibitory complex. Functionally, p23 maintained consistent antiplatelet efficacy in the complex matrix of human platelet-rich plasma. Flow cytometry demonstrated that p23 selectively suppresses integrin GPIIb/IIIa conformational activation without impairing P-selectin and CD63 surface expression. Crucially, p23 significantly reduced collagen-dependent three-dimensional thrombus formation in whole blood under biomimetic arterial flow, while still allowing for necessary residual platelet adhesion. Unlike direct integrin antagonists, p23 preserved physiological clot retraction and did not prolong plasma coagulation parameters. These findings validate the rational design of covalent PDI inhibitors and highlight peptide p23 as a promising candidate for preclinical in vivo evaluation, offering a favorable preliminary hemostatic profile and the potential for a reduced impact on hemostasis.

## 1. Introduction

Cardiovascular diseases are the leading cause of morbidity and mortality worldwide, with arterial thrombosis being a critical event in the pathophysiology of acute myocardial infarction and ischemic stroke [1,2]. Thrombus formation depends on a series of coordinated processes, including platelet activation, granule secretion, conformational changes in the GPIIb/IIIa receptor, and fibrin generation [3]. In this context, the search for new antithrombotic strategies constitutes a clinical and scientific challenge, particularly considering that current therapies, such as P2Y12 receptor antagonists, GPIIb/IIIa inhibitors, or PAR-1 antagonists, although proven effective, are associated with adverse effects such as an increased risk of bleeding or interindividual variability in response [4,5]. This has prompted the exploration of new molecular targets that allow for more selective modulation of platelet activation [6]. Indeed, rather than completely suppressing platelet function, selectively modulating specific signaling pathways represents a promising frontier in modern cardiovascular therapy to avoid bleeding complications [7].

Among these targets, protein disulfide isomerase (PDI) has emerged as a promising pharmacological target [8]. PDI is a thiol-disulfide oxidoreductase enzyme that belongs to the endoplasmic reticulum protein family, where it plays essential roles in protein folding through its oxidase, reductase, and isomerase activities [9,10]. However, PDI has also been shown to be exposed on platelet and endothelial surfaces during cell activation, where it participates in the regulation of key proteins for hemostasis and thrombosis, including adhesion receptors, integrins, and coagulation factors [11,12,13]. In vivo studies have confirmed that PDI inhibition reduces thrombus formation without significantly altering plasma coagulation parameters, reinforcing its relevance as a therapeutic target with a potentially improved safety profile compared to other antithrombotic agents [14,15].

PDI inhibition has been explored using different strategies, including small molecules such as PACMA31, quercetin, and isoquercetin, as well as natural derivatives with antioxidant and antithrombotic activity [16,17,18]. However, these compounds present limitations related to their bioavailability, selectivity, and potential toxicity [19]. In this context, synthetic peptides have established themselves as versatile tools in the biomedical field, as they allow the design of sequences with specific properties geared toward a molecular target [20,21]. Their high structural flexibility and the possibility of introducing controlled chemical modifications make them ideal candidates for exploring new inhibition strategies [22]. By leveraging deep learning architectures, generative models, and high-throughput computational screening, these technologies can rapidly explore vast and complex chemical spaces. They can predict key properties such as binding affinity, secondary structure, cell permeability, and metabolic stability before a candidate is synthesized in the laboratory. Models trained on large-scale proteomic and structural datasets can also support the de novo design of optimized peptide candidates, significantly reducing the number of synthesis-and-validation cycles while helping address traditional trade-offs in bioactivity [23].

The rational design of peptides capable of interacting with functional regions of PDI represents a novel strategy for the development of selective inhibitors [24]. Molecular docking studies and dynamic simulations allow prediction of the binding affinity and stability of these complexes, offering a robust platform for identifying candidates with therapeutic potential [25]. Subsequently, functional validation in platelet aggregation assays and thrombosis models is essential to establish their biological relevance.

Within this framework, the present study focuses on the rational design and characterization of novel peptides with antiplatelet activity mediated by extracellular PDI inhibition. A focused library of 34 peptides was initially designed by analyzing the evolutionary conservation of the PDI catalytic active site. From this comprehensive library, five strategically selected candidates were evaluated through standard and covalent molecular docking, alongside in vitro functional screening to assess their direct enzymatic inhibition and anti-aggregatory potential. This hierarchical workflow successfully identified a single lead candidate, peptide p23. The specific covalent inhibitory mechanism of p23 was subsequently characterized using robust MD simulations, while its ultimate translational efficacy and safety profile were validated through biomimetic microfluidic arterial thrombosis models and physiological hemostasis assays.

Unlike previous PDI-targeting scaffolds, the novelty of the present study lies in the integration of a comprehensive multi-scale pipeline combining sequence-based prediction using the ESM-2 (Evolutionary Scale Modeling) machine-learning model, covalent docking, MD simulations, and experimental ex vivo studies under physiologically relevant arterial flow conditions. This integrated approach allows the rational identification and characterization of peptide-based inhibitors using complementary computational and experimental strategies, extending beyond conventional screening approaches.

## 2. Results

### 2.1. Rational Design and Generation of the 34-Peptide Library and Machine Learning-Based Prediction of PDI-Interacting Peptides

**Figure 1:**
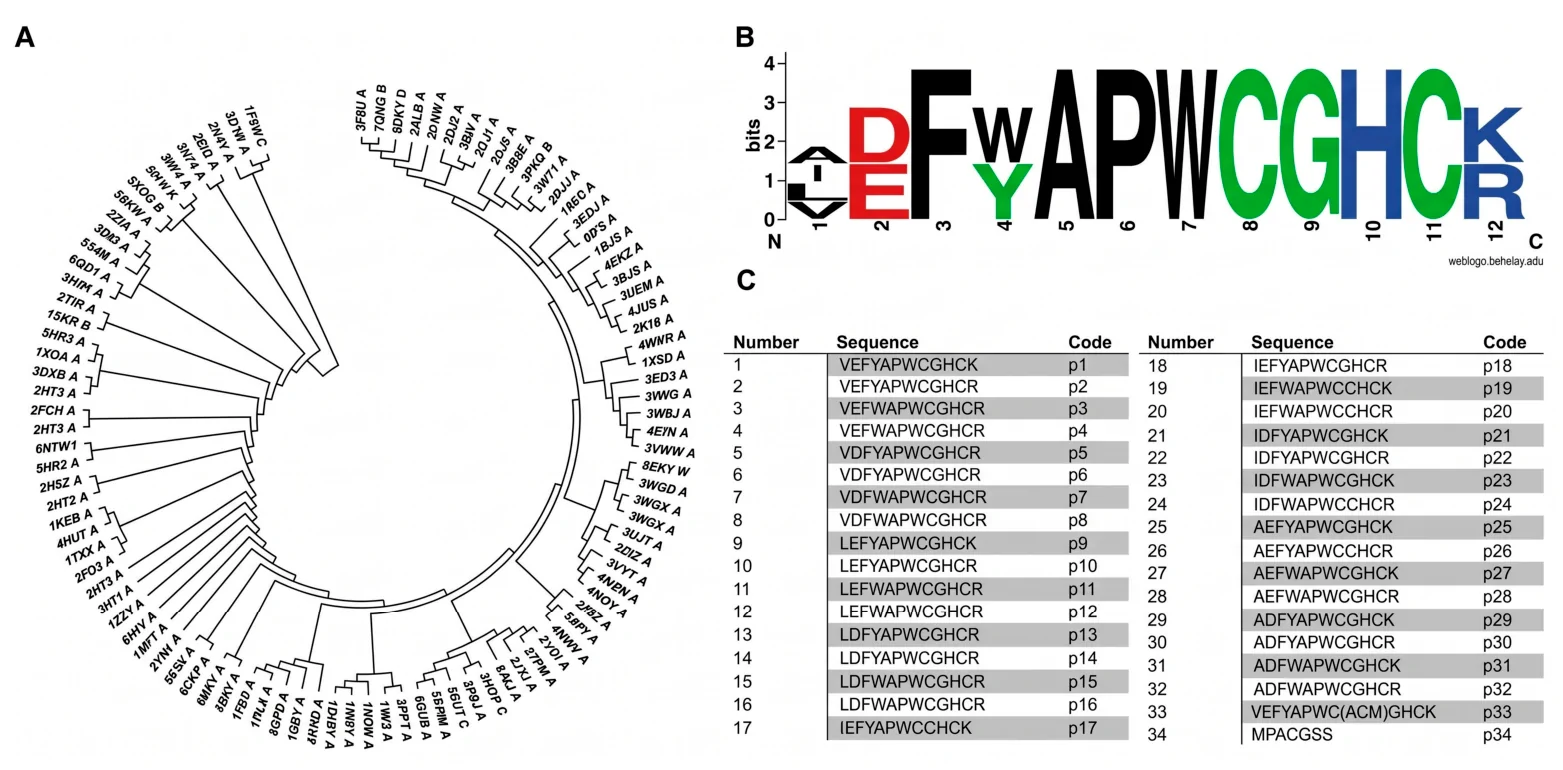
Rational design and characterization of the 34-peptide candidate library. (**A**) Circular dendrogram illustrating the structural homology relationships among various Protein Disulfide Isomerase (PDI) structures retrieved from thePDB. PDB accession codes are indicated for each structure. (**B**) WebLogo representation showing sequence conservation of the region surrounding the PDI catalytic active site. The overall height of each stack indicates the sequence conservation at that position, while the height of the letters within the stack reflects the relative frequency of the respective amino acid. Amino acids are colored according to their chemical properties: polar (green), basic (blue), acidic (red), and hydrophobic (black). The absolute conservation of the active CGHC motif (positions 8–11) is highlighted. (**C**) Summary table listing the amino acid sequences and code designations (p1 to p34) of all 34 peptides included in the initial screening library. (ACM) denotes acetamidomethyl cysteine protection in p33.Standard one-letter amino acid abbreviations used in this figure are: A, Alanine; C, Cysteine; D, Aspartic acid; E, Glutamic acid; F, Phenylalanine; G, Glycine; H, Histidine; I, Isoleucine; K, Lysine; L, Leucine; M, Methionine; N, Asparagine; P, Proline; Q, Glutamine; R, Arginine; S, Serine; T, Threonine; V, Valine; W, Tryptophan; Y, Tyrosine.

With the aim of developing specific peptide inhibitors for PDI, we initiated our study through a comprehensive bioinformatic analysis to characterize the structural environment of its catalytic sites. Structural homology analysis revealed a high degree of conservation among various PDIs deposited in the PDB (Protein Data Bank), organizing them into distinct structural families, as depicted in the circular dendrogram (Figure 1A).

Following this homologous alignment, we generated a WebLogo representation to visualize amino acid residue conservation in the regions adjacent to the redox catalytic motif. As shown in Figure 1B, the catalytic motif CGHC (positions 8–11) exhibits absolute conservation. Interestingly, the residues flanking this motif revealed critical conservation patterns, such as a bulky hydrophobic residue (Tryptophan, W5; Phenylalanine, F3) and a highly conserved Proline (P7). Simultaneously, we identified positions with greater evolutionary variability (positions 2, 4, and 12), which provide flexibility for introducing conservative substitutions without compromising the structural integrity of the interaction.

Integrating these evolutionary and structural findings, we applied a rational combinatorial approach to design a focused library of peptides with a high potential for interacting with the PDI active site. This process resulted in the generation of 31 novel peptides, where critical conserved residues were fixed, and positions identified as flexible were systematically varied. The final library was completed with a protected derivative of a previously reported peptide (p33) and a peptide reported with anticancer activity (p34), totaling 34 candidates. Their complete sequences and corresponding code designations are detailed in Figure 1C.

To streamline the screening of the 34-peptide library and rationally select candidates for empirical validation, we developed an ESM-2-based predictive model. The primary objective of implementing this model was twofold: first, to identify the most promising binding candidates, and second, to deliberately select peptides across a gradient of predicted affinities. This approach allows for the experimental validation of the model’s discriminatory power while exploring diverse structural motifs.

**Figure 2:**
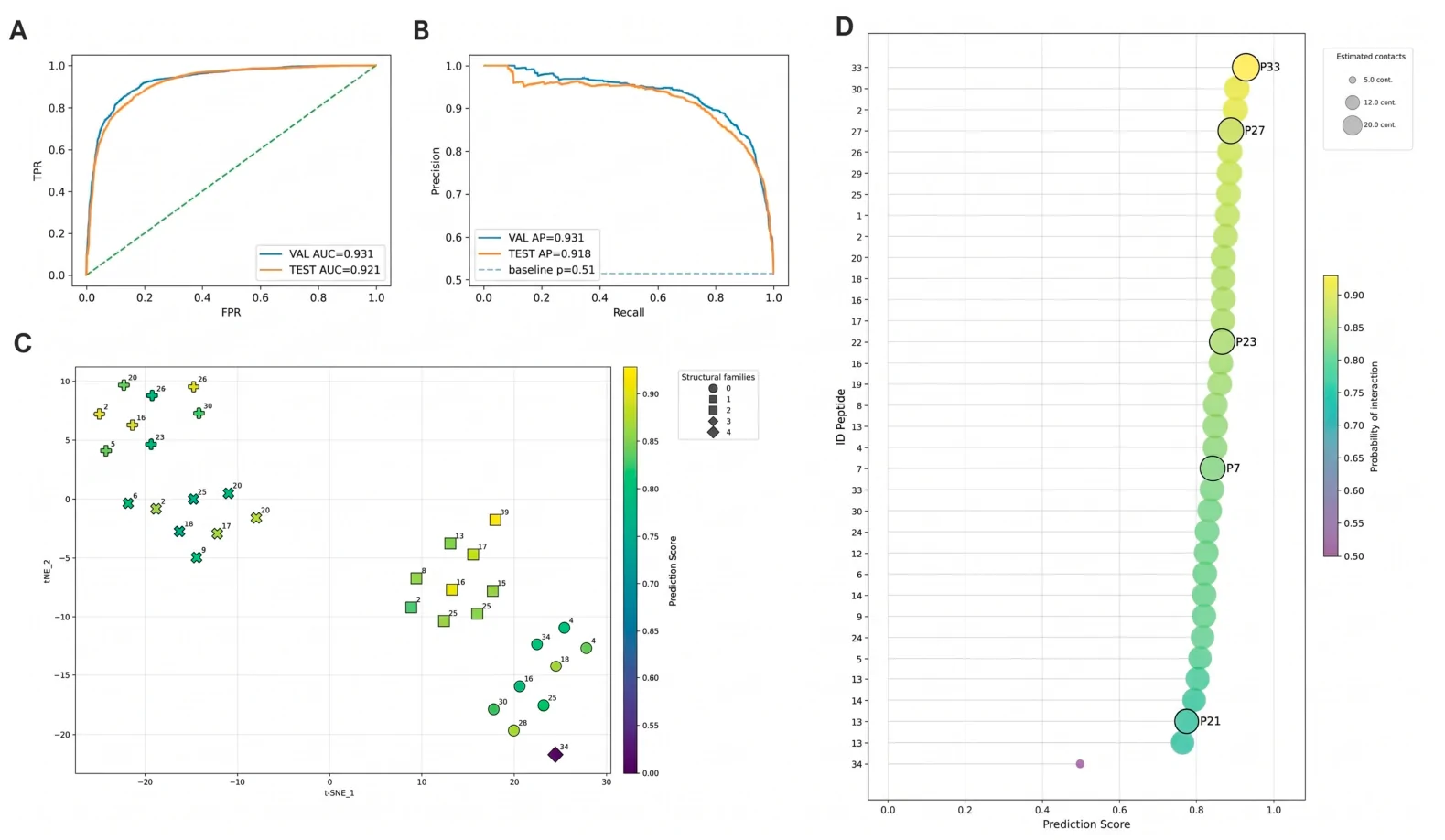
Machine learning-based prediction and rational selection of candidate peptides. (**A**) Receiver Operating Characteristic (ROC) curve and (**B**) Precision–Recall (PR) curve demonstrating the predictive performance of the ESM-2-based model on the validation (VAL) and independent test (TEST) sets. (**C**) t-SNE dimensionality reduction in the peptide library’s latent space, revealing five distinct structural families (clusters 0–4). Marker colors indicate the predicted interaction score. (**D**) Ranking of the 34 peptides based on their prediction score (x-axis) and probability of interaction (color gradient). Bubble size represents the estimated number of contacts. The five peptides strategically selected for in vitro validation (p7, p21, p23, p27, and p33), representing both the highest-affinity group and moderate-affinity candidates for model validation, are highlighted with black circles. The dashed line in (**A**) denotes a random model where the true positive rate equals the false positive rate.

The predictive performance of the ESM-2-based model was initially assessed using Receiver Operating Characteristic (ROC) and Precision–Recall (PR) analyses (Figure 2A,B). The model achieved an Area Under the Curve (AUC) of 0.931 in the validation set and 0.921 in the independent test set, indicating high reliability in distinguishing between strong and weak protein–peptide interactions. Furthermore, the Average Precision (AP) remained above 0.91 in both sets, significantly exceeding the baseline (*p* = 0.51), confirming the model’s robustness even with structural variations in the peptides.

To ensure that the selection process was not biased toward a single structural motif, we explored the latent space of the peptide library using t-SNE dimensionality reduction (Figure 2C). This analysis revealed five distinct structural families (clusters). Interestingly, peptides with high prediction scores were distributed across different groups, suggesting that multiple sequence-structure patterns may mediate effective PDI inhibition.

Based on these computational insights, five representative peptides (p7, p21, p23, p27, and p33) were strategically selected for further experimental assays. The rationale for this selection directly addresses the purpose of the predictive model (Figure 2D). First, to maximize the likelihood of finding potent inhibitors, we selected peptides from the highest-affinity prediction group (p33, p27, and p23), which exhibited interaction probabilities exceeding 0.85 and a high number of estimated contacts. Second, to experimentally validate the model’s accuracy and expand the structural diversity of our list of peptides, we deliberately included peptides with moderate-to-lower prediction scores belonging to different structural clusters (p7 and p21). By evaluating candidates across this predicted affinity gradient rather than focusing solely on the top-ranked cluster, we aimed to empirically confirm the model’s reliability and increase the probability of identifying inhibitors with distinct binding modes or enhanced pharmacological properties.

### 2.2. Structural Characterization of Candidate Peptide Binding Modes via Covalent and Non-Covalent Docking

The molecular interactions and binding orientations of the five strategically selected peptides (p7, p21, p23, p27, and p33) within the PDI catalytic active site were evaluated by performing both standard and covalent docking simulations targeting the critical redox-active Cys400 residue (Table 1).

Standard non-covalent docking was first employed to evaluate the initial accommodation of the peptides into the binding pocket prior to any chemical reaction. The results revealed that candidates p7, p21, p23, and p27 establish extensive interaction networks with the PDI catalytic domain, driven by specific contacts with key pocket residues. For instance, p23 forms a stable pre-reaction complex characterized by interactions with residues Asp363, Pro395, and Trp396. In this non-covalent state, the distances between the sulfur atom of PDI’s Cys400 and the sulfur atoms of the peptides’ cysteines ranged from 8.03 Å to 14.75 Å. Notably, peptide p33 failed to yield valid binding poses in either standard or covalent modalities. This was an expected outcome, as the bulky ACM protecting group on its cysteine likely introduces significant steric hindrance, preventing proper accommodation within the catalytic cleft and serving as a computational negative control.

Considering previous reports showing that modification of Cys400 can be relevant to PDI inhibition by different compounds [26,27], covalent docking was performed to model the transient disulfide bond formation between the free cysteines of the peptides and Cys400 of PDI. Among the evaluated candidates, p23 demonstrated highly favorable covalent binding parameters. While p21 exhibited the highest absolute covalent docking score (−9.0), p23 displayed the lowest (most stable) binding energy (−9.3 kcal/mol) and an optimal inter-sulfur distance of 2.20 Å involving its Cys11 residue. Peptides p7 and p27 also generated stable covalent poses, exhibiting inter-sulfur distances of 2.19 Å and 2.11 Å, respectively, albeit with distinct dihedral angles and less favorable energetic profiles compared to p23.

Having characterized the theoretical binding affinities and catalytic site occupancy of these diverse candidates, we next sought to determine whether these in silico parameters translate into functional biological activity. To this end, the five peptides were chemically synthesized to undergo empirical in vitro screening for direct PDI reductase inhibition and preliminary cellular safety.

### 2.3. In Vitro Screening of Candidate Peptides for PDI Reductase Inhibition and Cellular Safety

**Figure 3:**
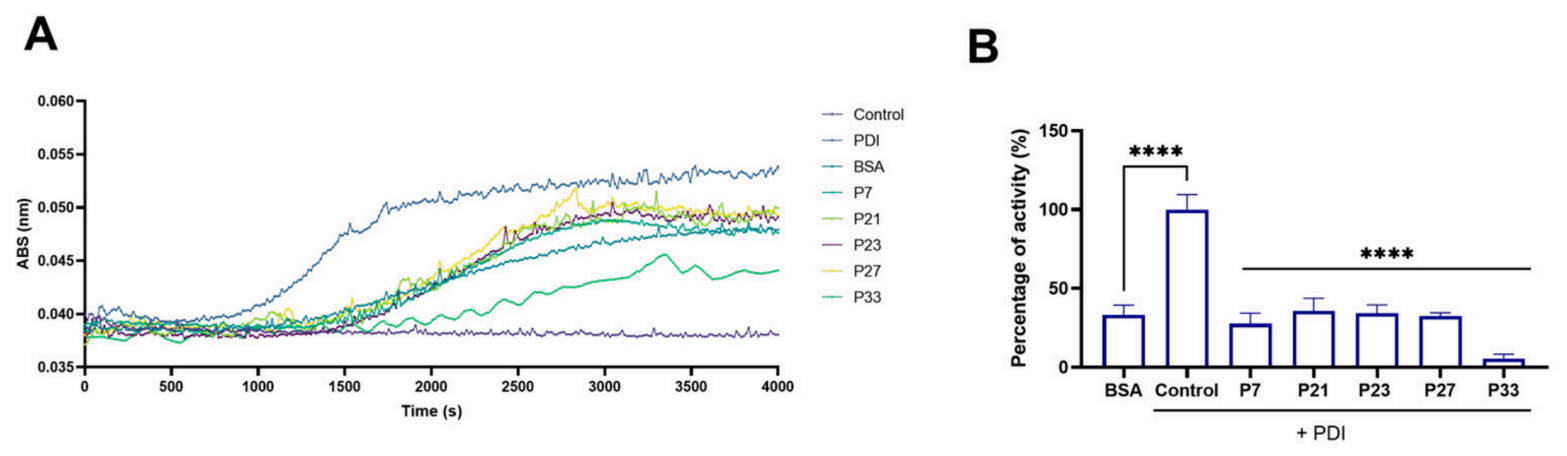
PDI reductase inhibitory activity of the candidate peptides. (**A**) Representative kinetic curves of PDI-mediated insulin reduction monitored by absorbance at 600 nm over time. (**B**) Percentage of PDI reductase activity in the presence of the candidate peptides (10 µM) relative to the uninhibited PDI control (100%). BSA (bovine serum albumin) was used as a negative background control. Data are expressed as mean ± SD of three independent experiments (*n* = 3). Statistical analysis was performed using one-way ANOVA (**** *p* < 0.0001 vs. Control + PDI).

The biological activity of the five synthesized candidate peptides (p7, p21, p23, p27, and p33) was empirically validated by first evaluating their ability to directly inhibit the enzymatic function of PDI using a standard insulin reduction assay. The reductase activity was continuously monitored by measuring the increase in absorbance at 600 nm due to the precipitation of reduced insulin B chains (Figure 3A). The in vitro screening revealed that the pre-incubation with the candidate peptides significantly impaired PDI function. Specifically, peptides p7, p21, p23, and p27 effectively reduced the PDI reductase activity to approximately 30–40% of the uninhibited control (**** *p* < 0.0001) (Figure 3B).Peptide p33 also showed a reduction in reductase activity. However, because p33 did not yield valid docking poses, likely due to steric hindrance caused by its protecting group, this biological effect suggests that p33 may inhibit PDI through an alternative non-covalent interaction mode or through assay-related mechanisms that are not captured by the spatial constraints of our in silico docking model.

**Figure 4:**
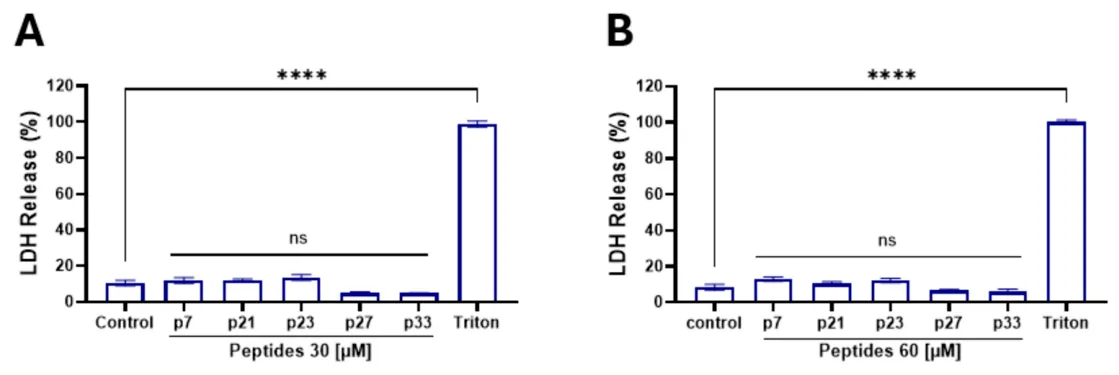
Cytotoxicity evaluation of the candidate peptides on human platelets. The cellular safety of the peptides was assessed by measuring LDH release from washed platelets after incubation at (**A**) 30 µM and (**B**) 60 µM peptide concentrations. Data are expressed as the percentage of LDH release relative to the maximum lysis control (Triton X-100), representing the mean ± SD of six independent experiments using blood from different healthy donors (*n* = 6). Statistical analysis was performed using one-way ANOVA followed by Tukey’s post hoc test (ns: not significant vs. Control; **** *p* < 0.0001 vs. Triton).

Concurrently, to ensure the physiological applicability of these candidates as potential antithrombotic agents, their preliminary cellular safety was assessed. An LDH (Lactate Dehydrogenase) release assay was performed on washed human platelets incubated with the peptides at 30 µM and 60 µM. Crucially, none of the five evaluated peptides induced significant cytotoxicity at either concentration. The LDH release levels remained statistically comparable to the basal resting state (ns, *p* > 0.05) and sharply contrasted with the maximum lysis control (Triton X-100, **** *p* < 0.0001) (Figure 4A,B). These results confirm that the candidate peptides possess a highly favorable safety profilein vitro, ensuring that subsequent functional antiplatelet effects are driven by specific pharmacological inhibition rather than nonspecific cellular toxicity.

### 2.4. Inhibition of Agonist-Induced Platelet Aggregation and Selection of the Lead Candidate

Having established the safety and target-specific enzymatic inhibition of the peptides, we performed a functional screening to evaluate their capacity to inhibit agonist-induced platelet aggregation. An initial screening was conducted at a fixed concentration of 60 µM using multiple physiological agonists (collagen, high-dose thrombin, and low-dose thrombin) in washed platelets, as well as collagen and ADP in platelet-rich plasma (PRP) (Supplementary Figure S1). The peptides showed no significant inhibitory effect against thrombin or ADP. However, peptides p7, p21, and p23 demonstrated a robust and significant inhibition of collagen-induced aggregation in washed platelets (**** *p* < 0.0001).

**Figure 5:**
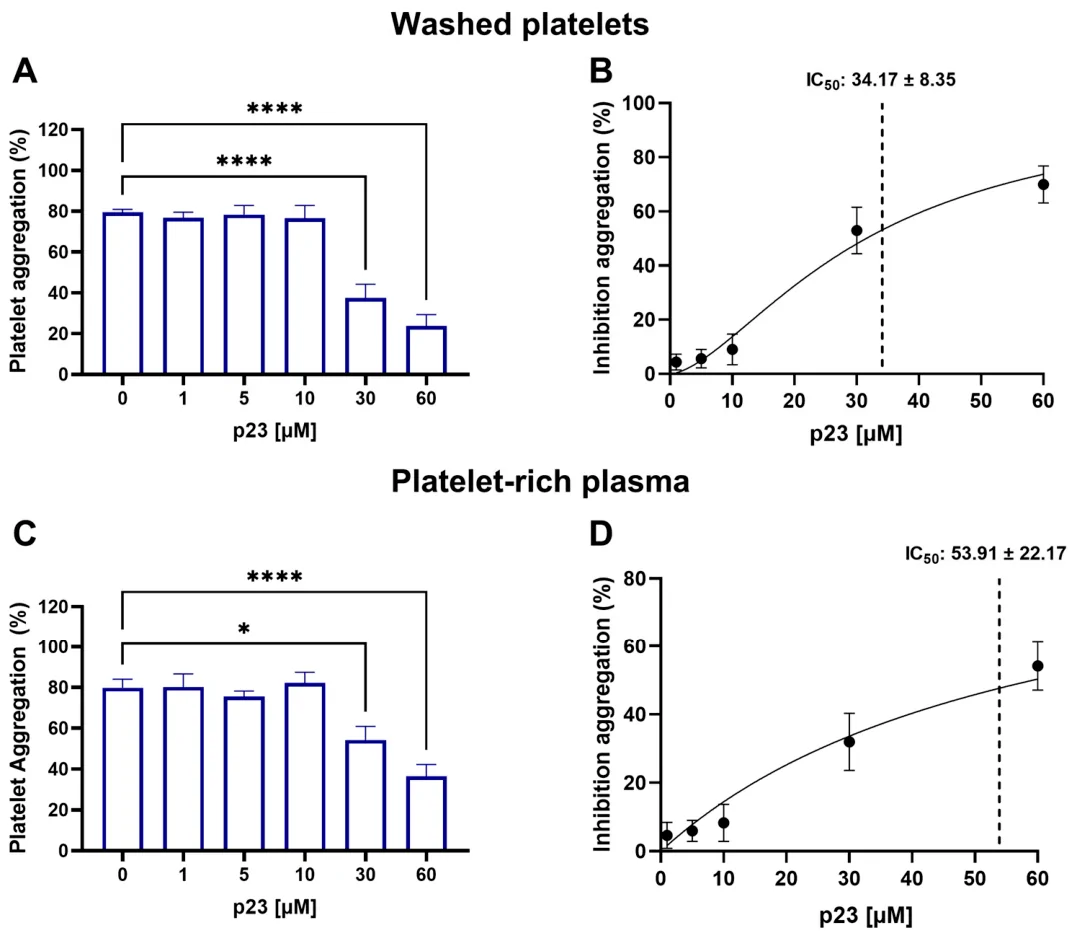
Inhibitory effect of p23 on collagen-induced platelet aggregation. The antiplatelet activity of p23 was evaluated in washed platelets (**A**,**B**) and platelet-rich plasma (PRP) (**C**,**D**) stimulated with 2 µg/mL of collagen. (**A**,**C**) Bar graphs representing the percentage of platelet aggregation at increasing concentrations of p23 (0–60 µM). Statistical significance was determined compared to the vehicle control (0 µM) (* *p* < 0.05, **** *p* < 0.0001). (**B**,**D**) Non-linear regression curves showing the percentage of aggregation inhibition used to calculate the half-maximal inhibitory concentration (IC_50_). In washed platelets, p23 exhibited an IC_50_ of 34.17 ± 8.35 µM (**B**), while in PRP, the IC_50_ was 53.91 ± 22.17 µM (**D**). Data are presented as mean ± SD of five independent experiments using blood from different healthy donors (*n* = 5).

To precisely quantify this efficacy, dose–response curves were generated for the active candidates. In washed platelets, peptides p7 and p21 exhibited half-maximal inhibitory concentrations (IC_50_) of 42.64 ± 17.89 µM and 41.27 ± 16.52 µM, respectively (Supplementary Figure S2, Table 2). Peptide p23 emerged as the most potent candidate in this experimental design, achieving an IC_50_of 34.17 ± 8.35 µM and significantly reducing aggregation at 30 µM and 60 µM (Figure 5A,B).

The definitive distinguishing factor among the candidates was revealed during the evaluation in PRP, a more complex physiological design containing plasma proteins. While p7 and p21 completely lost their inhibitory efficacy in PRP (IC_50_> 60 µM), peptide p23 successfully maintained significant antiplatelet activity (**** *p* < 0.0001 at 60 µM, * *p* < 0.05 at 30 µM) (Figure 5C). The calculated IC_50_ for p23 in PRP was 53.91 ± 22.17 µM (Figure 5D, Table 2).

Collectively, these functional screening results demonstrate that peptide p23 translates itsin silico binding profile and PDI reductase inhibition into a specific and safe antiplatelet efficacy in vitro, crucially retaining its efficacy in a complex physiological matrix rich in plasma proteins. Consequently, p23 was designated as the definitive lead candidate and was subjected to in-depth mechanistic characterization.

### 2.5. Atomistic Mechanism and Conformational Stability of the PDI-p23 Inhibitory Complex

The atomistic mechanism of inhibition and the structural stability of the p23–PDI interaction over time were evaluated through 500 ns MD simulations. Given the nature of PDI reductase inhibition, which requires the formation of a mixed disulfide bond, we simulated and compared both the pre-reaction (standard non-covalent) encounter complex and the post-reaction (covalent) stable inhibitory complex.

The trajectories revealed a distinct two-step binding mechanism. In the standard non-covalent simulation, p23 successfully maintained its general association with the PDI catalytic groove but exhibited expected structural flexibility. The distance between the reactive cysteines (PDI-C400 and p23-C11) fluctuated significantly between 10 and 25 Å, as the peptide dynamically explored the binding pocket before the chemical reaction (Supplementary Figure S4B). Consequently, the standard complex exhibited higher dynamic fluctuations, reflected in the RMSD of the overall system (Supplementary Figure S4B) and the elevated RMSF of both the PDI backbone and the p23 ligand (Supplementary Figure S3D,F).

Conversely, modeling the formation of the covalent disulfide bond revealed a profound stabilization of the entire system. Once anchored, the inter-sulfur interaction distance (C400-C11) remained rigidly locked at ~2 Å throughout the entire 500 ns trajectory (Supplementary Figure S4A). This covalent anchoring induced a systemic structural plateau: the RMSD of the PDI-p23 complex reached equilibrium rapidly and remained highly stable (Supplementary Figure S3A). Furthermore, the RMSF of the ligand was markedly reduced compared to the non-covalent state, indicating that the peptide’s conformation is effectively “frozen” and firmly accommodated upon binding (Supplementary Figure S3C,E).

**Figure 6:**
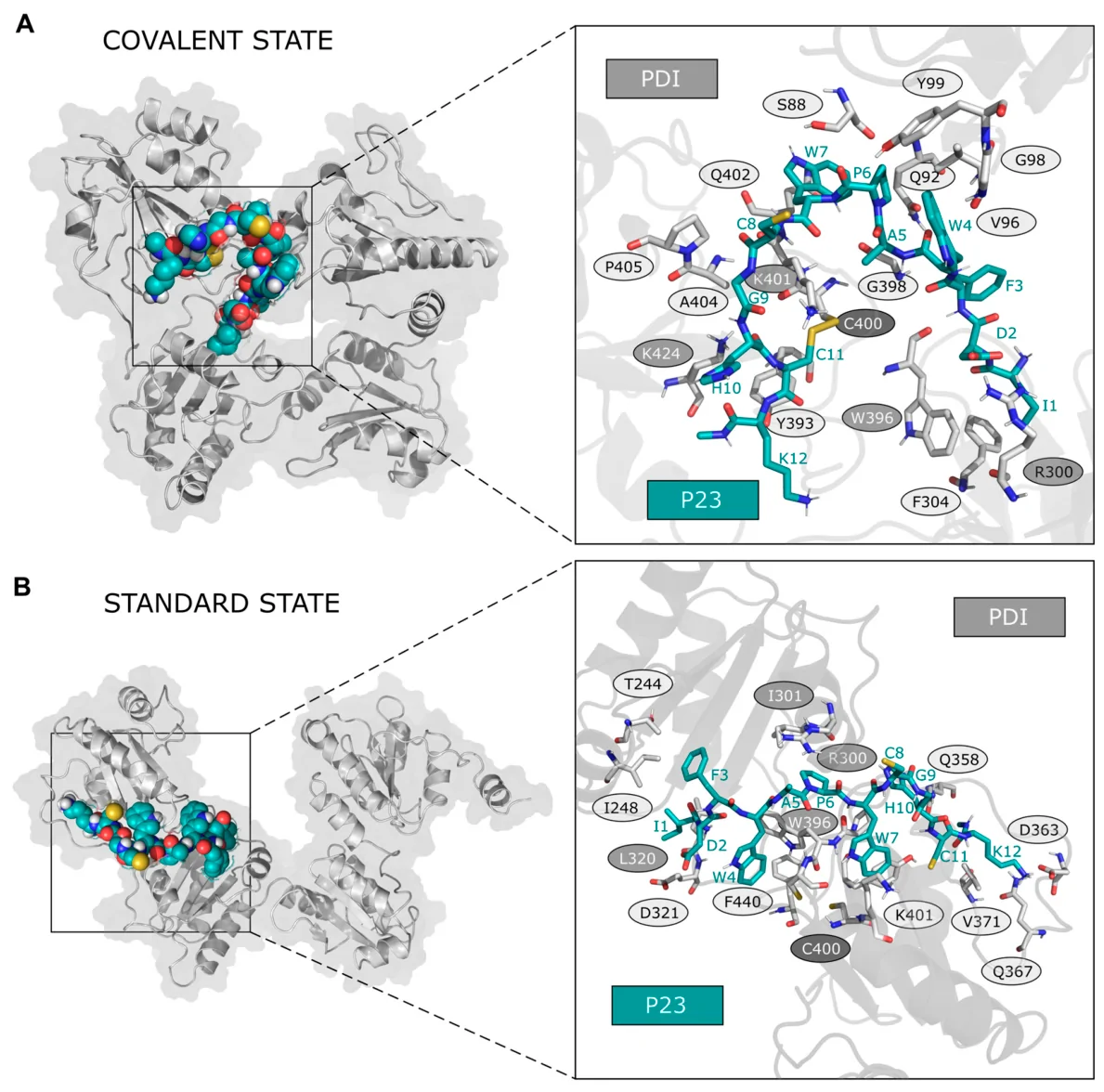
Atomistic resolution of the PDI-p23 complex in covalent and standard states. Representative 3D snapshots obtained from the 500 ns MD simulations with PDI shown in grey. (**A**) The top panels depict the post-reaction covalent state, highlighting the formation of the mixed disulfide bond between PDI-C400 and p23-C11. (**B**) The bottom panels display the pre-reaction standard state, where the reactive cysteines remain separated. In the zoomed-in views (right), the PDI residues located within a 4.5 Å radius of the peptide are indicated with oval labels. Residues establishing non-covalent interactions with p23 (cyan) are highlighted in dark grey, while the catalytic C400 residue is distinctly highlighted in light grey.

The enduring stability of the covalent complex is not solely dependent on the disulfide bond, but it is heavily supported by a persistent network of non-covalent interactions. Analysis of the non-bonded interaction energies (Coulomb + van der Waals forces) demonstrated a highly stable energetic profile over the 500 ns period for both states (Supplementary Figure S4C,D). Representative 3D snapshots of the MD trajectories (Figure 6) structurally confirm these quantitative findings. In the covalent state, p23 fits optimally within the PDI redox cleft, establishing stable contacts with critical surrounding residues within a 4.5 Å radius, which strongly support the maintenance of the inhibitory covalent bond at the active site. Taken together, the computational results (further visualized in Supplementary Videos S1 and S2) suggest that thein vitro functional findings for peptide p23 can be explained by forming the mixed disulfide bond at the PDI active site, creating a structurally locked inhibitory complex.

### 2.6. Peptide p23 Selectively Inhibits Integrin GPIIb/IIIa Activation Without Impairing Granule Secretion

Key membrane activation markers were evaluated using flow cytometry to elucidate cellular mechanisms by which PDI inhibition by p23 translates into impaired platelet aggregation. We specifically assessed the activation of integrin GPIIb/IIIa (via PAC-1 and Fibrinogen binding) and granule secretion (via P-selectin and CD63 expression) in both resting and collagen-stimulated washed platelets.

**Figure 7:**
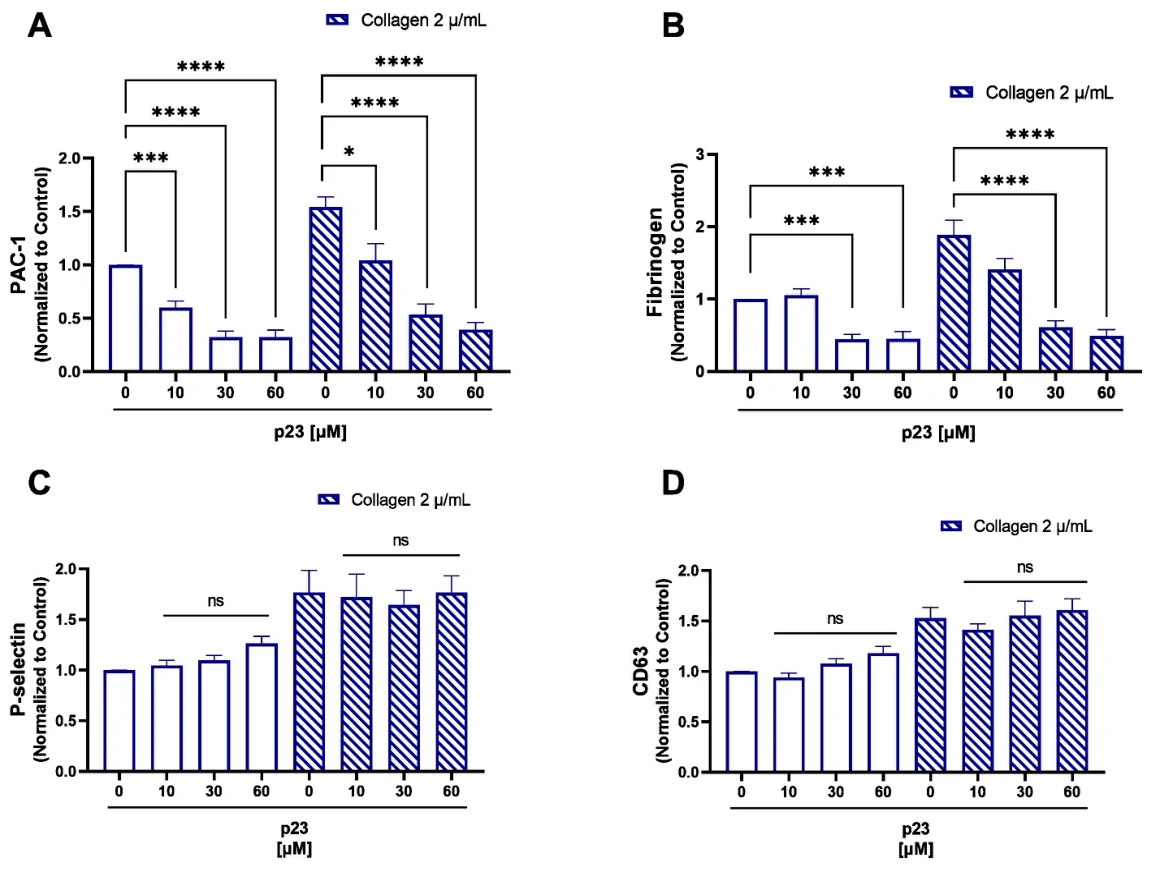
Peptide p23 inhibits GPIIb/IIIa integrin activation without affecting platelet granule secretion. Washed platelets were pre-incubated with vehicle or increasing concentrations of p23 (10, 30, and 60 µM) and evaluated in a resting state (unstimulated, white bars) or after stimulation with 2 µg/mL of collagen (light blue bars). Surface expression of specific activation markers was analyzed by flow cytometry. (**A**) PAC-1 binding, which reports the active conformation of the GPIIb/IIIa complex. (**B**) Fibrinogen binding to the platelet surface. (**C**) P-selectin surface expression, a marker for α-granule secretion. (**D**) CD63 surface expression, a marker for dense granule secretion. All data are expressed as mean values normalized to the resting control (0 µM p23, unstimulated) ± SD of seven independent experiments using blood from different healthy donors (*n* = 7). Statistical significance was determined using Two-way ANOVA followed by Bonferroni’s posthoc test comparing treatments to their respective vehicle control (* *p* < 0.05, *** *p* < 0.001, **** *p* < 0.0001, ns = not significant).

As expected, collagen stimulation significantly upregulated all activation markers in the vehicle control. Pre-incubation with peptide p23 resulted in a significant, dose-dependent reduction in PAC-1 and fibrinogen binding at 10, 30, and 60 µM (**** *p* < 0.0001 at the highest concentration), effectively blunting the integrin GPIIb/IIIa conformational change required for cross-linking and aggregation (Figure 7A,B). Interestingly, p23 also significantly reduced the basal activation levels of these markers in unstimulated platelets.

In sharp contrast, p23 did not significantly alter the surface expression of P-selectin (a marker of alpha-granule release) or CD63 (a marker of dense-granule release) at any of the tested concentrations, either in the resting state or following collagen stimulation (ns, *p* > 0.05) (Figure 7C,D). These findings demonstrate that p23 acts through a highly specific mechanism, selectively disrupting the inside-out signaling pathways leading to integrin GPIIb/IIIa activation without affecting global platelet degranulation mechanisms.

### 2.7. Peptide p23 Prevents In Vitro Thrombus Formation Under Biomimetic Flow While Preserving Physiological Hemostasis

**Figure 8:**
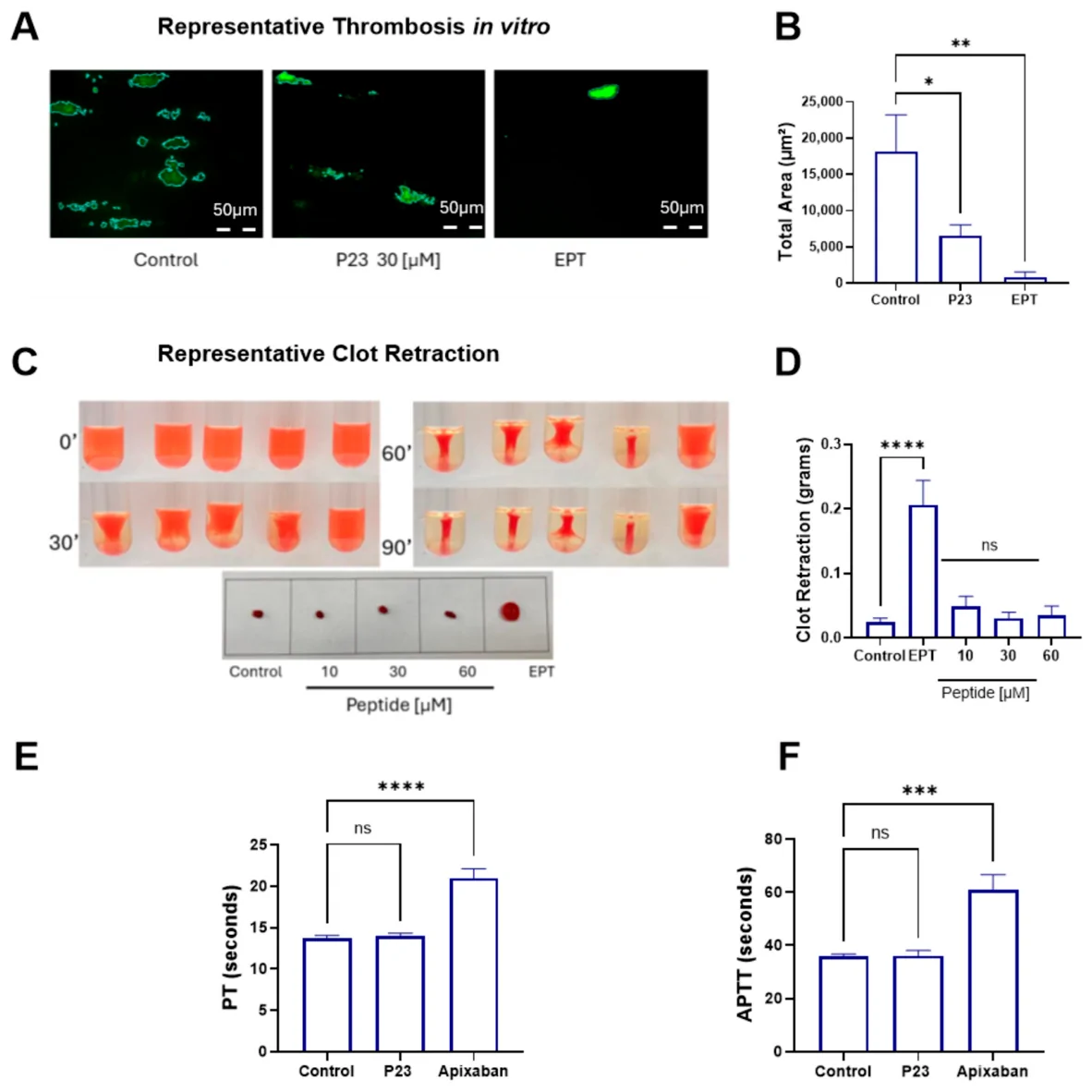
Peptide p23 selectively inhibits thrombus formation without affecting clot retraction and clotting times. (**A**) Representative fluorescence microscopy images of in vitro thrombus formation in human whole blood perfused over a collagen-coated surface. Platelets were fluorescently labeled and treated with either vehicle (Control) or 30 µM of p23. (**B**) Quantification of total thrombus area (µm^2^). (**C**) Representative macroscopic images of the clot retraction process over 90 min and the final isolated clots. (**D**) Quantification of clot retraction expressed as the residual weight (grams) of the clots after 90 min. Eptifibatide (EPT) was used as a positive control for retraction inhibition. (**E**) Prothrombin Time (PT) and (**F**) Activated Partial Thromboplastin Time (APTT) in human plasma treated with vehicle, p23, or Apixaban (positive control). All data are presented as mean ± SD of independent experiments using blood from different healthy donors (in vitro thrombosis *n* = 7; clot retraction *n* = 8; PT and APTT *n* = 6). Statistical significance was determined using One-way ANOVA compared to the respective control (* *p* < 0.05, ** *p* < 0.01, *** *p* < 0.001, **** *p* < 0.0001, ns = not significant).

The antithrombotic efficacy of the lead candidate under physiological hemodynamic conditions was confirmed by evaluating thrombus formation using a microfluidic perfusion system over a collagen-coated surface at arterial shear stress. While the vehicle control blood formed large, stable platelet aggregates, pre-incubation with p23 (at 30 µM) significantly reduced thrombus formation without inducing the complete abolition of aggregates observed with the clinical integrin inhibitor eptifibatide (Figure 8A, Supplementary Videos S3 and S4).Quantitative analysis confirmed that p23 significantly decreased the total thrombus area (* *p* < 0.05) (Figure 8B).

A major challenge in antithrombotic development is the increased risk of bleeding and impaired wound healing. Therefore, we assessed the impact of p23 on clot retraction, a late phase of hemostasis critically dependent on functional integrin GPIIb/IIIa outside-in signaling, and plasma coagulation cascades. Macroscopic observation and final clot weight quantification revealed that, unlike eptifibatide (which completely abolished clot retraction, **** *p* < 0.0001), p23 (10–60 µM) did not impair this process. The final clot weights in the p23-treated samples were statistically indistinguishable from the vehicle control (ns, *p* > 0.05) (Figure 8C,D).

Finally, standard coagulation assays were performed in platelet-poor plasma to rule out nonspecific interference with the coagulation cascade. Peptide p23 (at 30 µM) did not prolong the Prothrombin Time (PT) or the Activated Partial Thromboplastin Time (APTT) compared to the vehicle control (ns, *p* > 0.05), whereas the clinical anticoagulant apixaban significantly increased both parameters (Figure 8E,F). Collectively, these results indicate that p23 exerts a potent antithrombotic effect under arterial flow, while preserving the secondary hemostatic pathways and clot structural consolidation, suggesting a favorable safety profile.

## 3. Discussion

Despite advances in antithrombotic therapy, the development of novel agents that effectively prevent pathological vessel occlusion without elevating the risk of bleeding remains a primary challenge in cardiovascular pharmacology [5,28]. Over the last decade, extracellular PDI has emerged as a crucial mediator of platelet activation and thrombus propagation, positioning it as a highly attractive and validated antithrombotic target [12,14]. In this study, utilizing a rational, bioinformatics-driven peptide design approach [24] targeting the conserved regions of the PDI active site, we identified peptide p23 as a potent, specific, and safe PDI reductase inhibitor. Our functional and physiological findings demonstrate that p23 selectively disrupts integrin GPIIb/IIIa activation that inhibits platelet aggregation, and prevents thrombus formation dependent on platelet–platelet interactions. Notably, it achieves this significant antithrombotic effect without compromising global platelet degranulation, impairing physiological clot retraction, or interfering with the plasma coagulation cascade, thereby suggesting a favorable preliminary hemostatic profile.

The biological efficacy of p23 is fundamentally rooted in its unique atomistic mechanism of interaction within the PDI catalytic cleft. Our MD simulations revealed a defined two-step binding process: an initial dynamic encounter state that allows the peptide to navigate the binding pocket, followed by a profound structural stabilization upon the formation of a mixed disulfide bond between p23-Cys11. Once this covalent anchor is established, the conformational flexibility of the ligand is drastically restricted, effectively “locking” the peptide within the redox cleft. Bioinformatic data suggest that p23 can also react with the critical catalytic PDI Cys400. The simulation results suggest a mechanism of active-site stabilization that is consistent with the pharmacological behavior of other characterized PDI inhibitors. These include previously reported modifications of Cys400 by targeted peptides that exhibit covalent binding to the C-terminal CGHC redox motif [26], as well as by molecules such as nitroarachidonic acid, which impair the enzyme’s reductase activity through the reversible formation of covalent adducts with critical cysteine residues [27].

The translation of in vitro enzymatic inhibitors into physiological environments often represents a major bottleneck in drug development, particularly for peptide-based therapeutics, which are prone to proteolytic degradation or non-specific sequestration by plasma proteins. In our functional screening, while p7 and p21 exhibited promising antiplatelet activity in washed platelets, their inhibitory efficacy was completely abolished in platelet-rich plasma (PRP) (IC_50_ > 60 µM). In sharp contrast, p23 successfully maintained significant antiplatelet activity in PRP, highlighting superior bioavailability and structural robustness. This differential efficacy suggests that the specific sequence and conformational arrangement of p23 not only ensure optimal target engagement at the PDI catalytic site but also confer resistance against the complex interfering matrix of human plasma. Retaining functionality in PRP is a critical prerequisite for systemic administration, positioning p23 as a promising candidate for future in vivo studies. The p23 peptide contains a CGHC motif, and the cysteine residues are expected to be most active in their reduced state, which may be required for the proposed inhibitory activity. Thus, oxidation of these cysteines could reduce the peptide’s capacity to interact with PDI and diminish its inhibitory effect. Although the complex redox environment of plasma could potentially promote oxidation of the cysteine residues, the inhibitory activity of p23 is retained in both PRP and whole blood, suggesting that a sufficient fraction of the peptide remains in an active redox state under these conditions. Nevertheless, the reduced inhibitory potency observed in PRP compared with washed platelets may indicate that plasma components influence the redox state, stability, or availability of p23. Importantly, the CGHC motif should not necessarily be considered the sole or main determinant of p23 activity, as other residues within the peptide may contribute to PDI recognition and inhibition.

Beyond systemic stability, the clinical translation of novel antiplatelet agents relies heavily on their capacity to inhibit thrombosis without inducing broad cellular dysfunction [29]. Our flow cytometry analysis revealed a highly specialized mechanism of action: p23 dose-dependently suppressed the conformational activation of integrin GPIIb/IIIa (evidenced by reduced PAC-1 and fibrinogen binding) without altering the surface expression of P-selectin or CD63. Extracellular PDI is known to be strictly required for the final steps of the inside-out signaling cascade that triggers the high-affinity state of integrin GPIIb/IIIa [30]. By selectively inhibiting PDI, p23 effectively uncoupled the integrin activation pathway from the global degranulation response. Consequently, platelets treated with p23 are rendered incapable of forming the stable fibrinogen cross-links required for aggregation, yet they retain their ability to release alpha and dense granules. This selective interference is highly desirable, as it prevents pathological vessel occlusion while preserving the secretion of granule contents that are vital for localized tissue repair and immune modulation [31].

A significant limitation of the present study is the lack of experimental confirmation of the formation of a mixed disulfide bond between p23 and relevant cysteine residues necessary for PDI activity; the proposal of covalent interaction is currently supported by computational predictions and indirect functional assays. Although the atomistic simulations provide strong support for a potential covalent anchoring mechanism, definitive experimental evidence demonstrating the formation of the p23–PDI covalent adduct is still lacking. Experimental approaches, such as liquid chromatography–tandem mass spectrometry (LC-MS/MS) to directly detect the adduct or site-directed mutagenesis of PDI, i.e., C400A, to assess the functional relevance of this residue, will be necessary to validate this proposed mechanism. Therefore, confirming the covalent nature and residue-specificity of this interaction in vitro represents an important objective for future investigations.

The ultimate physiological validation of p23 lies in its profound antithrombotic efficacy under arterial shear conditions coupled with its negligible impact on fundamental hemostatic safety markers. Currently, direct integrin GPIIb/IIIa antagonists, such as eptifibatide, are used in acute clinical settings because of their potent anti-aggregatory effects [32]. However, their use is severely limited by a high risk of life-threatening hemorrhage, largely driven by the complete blockade of integrin outside-in signaling, which abolishes physiological clot retraction and destabilizes hemostatic plugs. Importantly, while p23 demonstrated significant antithrombotic efficacy under biomimetic microfluidic flow, it did not alter the clot retraction process. Although it might seem counterintuitive that p23 prevents soluble fibrinogen binding to GPIIb/IIIa without impairing clot retraction, this discrepancy is consistent with the understanding that the integrin binding sites for soluble fibrinogen (essential for initial aggregation) and polymerized fibrin (essential for clot retraction) are functionally distinct. Thrombin-induced fibrin polymerization exposes novel binding epitopes that generate high-avidity interactions, sustaining tension transmission and clot retraction even when soluble fibrinogen binding is suppressed. Furthermore, p23 did not increase standard coagulation parameters (PT and APTT), indicating an absence of cross-reactivity with the plasma coagulation cascade. By targeting the upstream regulatory function of extracellular PDI rather than functioning as a direct integrin inhibitor, p23 effectively circumvents the severe hemostatic impairment associated with current therapies, suggesting a potential for a reduced impact on physiological hemostasis, though comprehensive in vivo validation remains necessary to fully establish its safety profile.

## 4. Materials and Methods

### 4.1. Peptide Design

The peptide library design was based on a peptide previously reported as a protein disulfide isomerase (PDI) inhibitor [26], designated as Peptide 1.To identify conserved regions in PDI, the protein’s FASTA sequence (PDB code: 4EKZ) was obtained from the PDB [33]. A BLASTpv2.16.0 [34] alignment was then performed using the structures deposited in the PDB as a database to retrieve homologous sequences of PDIs from different species.

The selected sequences were aligned using Clustal Omega v1.2.4 software [35]. To evaluate the evolutionary relationships among the homologous PDIs, a phylogenetic tree was constructed using MEGA v12.0.11 software [36]. The evolutionary history was inferred using the Maximum Likelihood method, with topological robustness assessed via 1000 bootstrap replicates (Figure 1A). Additionally, the alignment results were graphically represented in WebLogo v3.9.0 [37]. This allowed the conservation of residues at different positions to be visualized, particularly within domains containing the CGHC catalytic motif (Figure 1B). Through this process, it was possible to identify both the conserved and the most variable amino acids within the redox region of PDI.

Based on these characteristics, a rational combinatorial approach was applied to generate a total of 31 different peptides. This approach introduced variations at strategic positions, maintaining the conservation of critical residues while modifying those with greater evolutionary flexibility.

- Peptide 33: Corresponds to Peptide 1, but features a protecting group on one of its cysteines.
- Peptide 34: is a peptide previously reported to have anticancer activity, isolated from a variety of *Phaseolus vulgaris* [38].

In total, the library consisted of 34 peptides (Figure 1C), designed and selected based on structural conservation criteria, potential for interaction with the catalytic site, and sequence diversity.

### 4.2. Training Dataset Construction and Curation

To develop the predictive models, a robust dataset of protein–peptide interactions with structural resolution was assembled. The PepBDB [39] database was selected, as it integrates high-resolution complexes derived from the PDB.

The extraction and curation processes were automated using a custom pipeline developed in Python v3.14, utilizing the Biopython library for structural manipulation. The main steps included quality filtering, where only structures with defined crystallographic resolution and complete interface data were selected.

Biological sequences were reconstructed from structural files, translating non-standard amino acids into their natural equivalents to ensure the chemical consistency of the model.

A geometric contact analysis was implemented using a neighbor search algorithm (NeighborSearch), with a cutoff radius of 4.5 Å between heavy atoms. The resulting number of contacts served as a proxy for interaction affinity.

### 4.3. Model Architecture and Sequence Representation (ESM-2)

Instead of traditional physicochemical descriptors, a transfer learning approach was employed using the ESM-2 (Evolutionary Scale Modeling) [40] protein language model, specifically the esm2_t6_8M_UR50D variant.

This architecture captures complex semantic and evolutionary representations of the sequences. This model features an embedding generation process, where peptide and protein sequences were processed to extract vectors from the model’s final representation layer.

An average clustering technique was applied along the sequence dimension to obtain a fixed-length vector embedding for each input. Furthermore, the peptide and protein embeddings were concatenated to feed a logistic regression classifier. This model was trained to predict the probability of a “strong interaction” based on the latent space generated by ESM-2.

### 4.4. Training Strategy and Performance Evaluation

For supervised training, the contact variable was transformed into a binary label using the median of the dataset (P50) as a dynamic threshold. Complexes with a contact density above the 50th percentile were classified as positive interactions.

The robustness of the model was ensured through several validation stages. The first stage consisted of group-based validation, where the dataset was divided into training (60%), validation (20%), and test (20%) sets using a GroupShuffleSplit strategy, as implemented in the Scikit-learn library [41]. This ensured that interactions associated with the same PDB ID were not shared between sets, preventing overfitting due to structural homology.

The second stage involved performance metrics, where the model’s discriminative capacity was evaluated by calculating the Area Under the ROC Curve (AUC-ROC) and Average Precision (AP) on both the validation and independent test sets.

The third stage was latent space clustering, in which unsupervised validation was performed using K-Means [42] clustering and t-SNE [43] dimensionality reduction on the high-affinity peptide embeddings. This analysis allowed for the identification of structural families with potential inhibitory activity.

After data curation, the final dataset comprised 13,154 protein–peptide complexes, consisting of 6766 positive (strong interaction) and 6388 negative (weak interaction) examples. The dataset was divided into training (7871 complexes), validation (2629 complexes), and test (2654 complexes) sets. To ensure full reproducibility and facilitate community access, the complete computational pipeline, including data curation, ESM-2 embedding generation, model training, and unsupervised clustering scripts, is open-source and publicly available in the GitHub repository (version 1.0.0): https://github.com/franroca15/pdi-peptide-ml-prediction (accessed on 19 August 2026).

### 4.5. Structural Construction and Peptide Preparation

The three-dimensional structures of the 34 peptides were generated using the AlphaFold Server [44], incorporating modifications at the N- and C-termini by adding an acetyl group (Ac-) and an amidation (-amide), respectively, to stabilize the conformation and simulate physiological conditions.

Before docking studies, the peptide structures were subjected to energy minimization in Maestro Schrödinger Release 2021-3 software [45], using the OPLS3e force field [46]. This procedure reduced conformational strains and optimized the geometry of the peptides for subsequent analysis of their interaction with the target protein.

### 4.6. Molecular Docking

Molecular docking studies were performed for peptides in the library using two complementary approaches implemented in the Schrödinger Suite [45].

First, to evaluate the formation of a stable inhibitory complex, covalent docking was executed using the CovDock [47] module. This analysis was specifically targeted at Cys400, the resolving cysteine within the a’ catalytic domain (PDB code: 4EKZ). The a’ domain was strategically selected over the a domain because it operates in close structural synergy with the adjacent b’ domain (the principal substrate-binding site of PDI). This b’-a’ architecture forms the primary interface required for the recognition and reduction in macro-substrates critically involved in platelet activation [48].

Then, a standard (non-covalent) docking protocol was performed using Glide [49] to provide a comprehensive assessment of the binding affinity and orientation of the peptides at the catalytic site under physiological-like conditions.

### 4.7. Peptide Synthesis

The peptides were synthesized by solid-phase peptide synthesis using the Fmoc/tBu strategy at the Laboratory of Peptide Synthesis (Curauma Biotechnology Center, Pontificia Universidad Católica de Valparaíso, Valparaíso, Chile). The peptides were subsequently purified by high-performance liquid chromatography (HPLC), and their identities were confirmed by mass spectrometry (MS). The minimum purity accepted for experimental use was 95% [50].

### 4.8. Preparation of PRP and Washed Platelets

Blood samples were obtained from volunteer donors who had not received antiplatelet medication for at least 10 days. All participants signed an informed consent form, approved by the Scientific Ethics Committee of the University of Talca (Protocol N° 29-2021) [51,52].

Blood was collected in tubes containing acid-citrate-dextrose (ACD) in a 4:1 *v*/*v* ratio (blood:ACD). It was then centrifuged for 12 min at 250× *g* to obtain platelet-rich plasma (PRP).

To obtain washed platelets, the PRP was centrifuged again for 8 min at 900× *g* to obtain the platelet pellet. The plasma supernatant was removed, and the platelets were resuspended in calcium-free Tyrode’s buffer supplemented with ACD (5:1 *v*/*v*). A second centrifugation was then performed for 8 min at 900× *g*, after which the pellet was resuspended in calcium-free Tyrode’s buffer [53].

Platelet concentration was determined using a hematology counter (Mindray BC-3000 Plus; Mindray, Shenzhen, China) and adjusted to the desired value. The washed platelet suspensions were used within 3 h of collection [54].

### 4.9. Cytotoxicity Assay

Platelet cytotoxicity was evaluated using a commercial LDH Cytotoxicity Kit (Cayman Chemical, Ann Arbor, MI, USA) according to a modified protocol [55]. Briefly, washed platelets (250 μL) were incubated with the synthetic peptides (p7, p21, p23, p27, and p33) at final concentrations of 30 μM and 60 μM, and with their respective controls, for 10 min at 37 °C. Triton X-100 (10%) was utilized as the maximum lysis control (100% cytotoxicity). Following incubation, the mixtures were centrifuged at 3000 rpm for 8 min at 4 °C. Subsequently, 25 μL of the supernatant was collected and transferred to a 96-well microplate, followed by the addition of 25 μL of the LDH reaction solution (containing 1% *v*/*v* each of NAD^+^, lactate, diaphorase, and INT reconstituted in the kit’s assay buffer). The microplate was incubated for 30 min at 37 °C under constant orbital shaking. Finally, absorbance was measured at 490 nm using a microplate reader, and the percentage of cytotoxicity was calculated relative to the maximum lysis control [56].

### 4.10. Reductase Activity of PDI with Peptides

Protein disulfide isomerase reductase activity was assessed using the standard insulin reduction assay. The standard reaction was prepared with recombinant PDI (1.2 µM), dithiothreitol (DTT, 1.2 mM), and insulin (1 mg/mL) in the appropriate buffer. Negative controls included conditions without insulin, as well as a control with bovine serum albumin (BSA, 1.2 µM). To evaluate the effect of peptides on reductase activity, they were added to the reaction at a final concentration of 10 µM before the addition of insulin.

The reaction was initiated by adding insulin to the system previously mixed with PDI, DTT, and the peptides, and the increase in turbidity due to the precipitation of reduced insulin B chains was monitored. Absorbance was recorded at 600 nm for 60 min in a Shimadzu UV/Vis spectrophotometer (Shimadzu, Kyoto, Japan).

Reductase activity was determined from the initial slope of the absorbance increase (ΔAbs_600_nm/Δt), which reflects the ability of PDI to catalyze insulin reduction in the presence or absence of peptides [57].

### 4.11. Platelet Aggregation Assays

Washed platelets (200–250 × 10^9^ platelets/L), in the presence of 2 mM CaCl_2_, were incubated with the peptides for 5 min at 37 °C. Then, platelet aggregation was initiated by the addition of the physiological agonist thrombin(0.01 and 0.1 U/mL). In the case of PRP, aggregation was induced using collagen (2 µg/mL) or ADP (4 µM). Reactions were carried out in a 96-well microplate, maintained at 37 °C with constant shaking at 1000 rpm [55]. Platelet aggregation was assessed by measuring absorbance changes at 405 nm on an Epoch spectrophotometer (BioTek Instruments, Winooski, VT, USA). For peptides that showed more than 50% inhibition of aggregation, the half-maximal inhibitory concentration (IC_50_), defined as the concentration of the peptide required to reduce the maximal agonist-induced platelet aggregation by 50% relative to the uninhibited vehicle control, was calculated by non-linear regression analysis [58].

### 4.12. Molecular Simulations

For the MD studies, the best standard (non-covalent) and covalent docking poses of the lead candidate, peptide p23, interacting with Cys400 of the PDI active site were selected as starting coordinates. Both protein–peptide structures were prepared in Maestro Schrödinger Release2021-3 [45] using the Protein Preparation Wizard and Ligand Preparation modules, ensuring correct protonation states and geometric optimization of the complexes.

Subsequently, each simulation system was generated in an orthorhombic box utilizing a TIP3P water model. The systems were neutralized by the addition of sodium ions (Na^+^) and supplemented with a salt concentration of 0.15 M NaCl.

The initial preparation for both systems included a 30,000-step energy minimization, followed by a 10 ns equilibration under an isothermal-isobaric (NPT) ensemble at 300 K and 1 atm. Production simulations were then carried out for 500 ns under NPT ensemble conditions (300 K, 1 atm). Trajectories were recorded for subsequent analysis of the conformational stability and dynamics of the protein–peptide interactions in both the standard and covalent states.

### 4.13. Platelet Activation Markers

Washed platelets (200–250 × 10^6^ platelets/mL) were pre-incubated with peptide p23 at concentrations of 10, 30, and 60 μM, followed by activation with collagen (2 μg/mL). To evaluate membrane activation markers, aliquots from each condition were collected and distributed into separate tubes for dual-color staining using two fluorophores (FITC and PE). Samples were labeled with the following specific antibodies: Anti-Human PAC-1 and Anti-Human Fibrinogen conjugated to FITC, and Anti-Human CD62P and Anti-Human CD63 conjugated to PE (BD Biosciences, San José, CA, USA; Sigma-Aldrich, St. Louis, MO, USA). The platelet population was gated and identified based on its characteristic forward (FSC) and side (SSC) scatter profiles. Fluorescence signals were acquired using a BD FACSLyric flow cytometer (BD Biosciences, USA), and data were analyzed to determine the mean fluorescence intensity (MFI) for each marker and normalized to the resting control [52,59,60].

### 4.14. Clot Retraction Assay

Clot retraction was performed using platelet-rich plasma (PRP) supplemented with red blood cells to facilitate visualization and quantification [61]. Briefly, venous blood was collected in 3.2% sodium citrate tubes and centrifuged at 1200 rpm (250× *g*) for 12 min to obtain PRP. In glass tubes, 750 μL of calcium-free Tyrode’s buffer was mixed with 200 μL of PRP and 5 μL of red blood cells. The mixtures were pre-incubated for 10 min at room temperature with peptide p23 (10, 30, and 60 μM), vehicle (positive control), or eptifibatide (10 μM) as a negative control. Coagulation was initiated by adding 50 μL of thrombin (final concentration: 0.5 U/mL), and a sealed glass capillary tube was placed in the center of each tube to serve as a support for the retracting clot. The reaction was allowed to proceed for 90 min at 37 °C. High-resolution photographs were captured at baseline (0 min) and at 30, 60, and 90 min. At the end of the incubation period, each clot was carefully removed, weighed, and recorded to quantify the extent of clot retraction [62].

### 4.15. Blood Clotting Times

Prothrombin time (PT) and activated partial thromboplastin time (aPTT) assays were performed using an automated coagulation analyzer. Briefly, venous blood was collected into tubes containing 3.2% sodium citrate and centrifuged for 15 min at 2500× *g* to obtain platelet-poor plasma (PPP). To evaluate the effect on coagulation times, PPP was pre-incubated with peptide p23 at a final concentration of 30 μM or with the vehicle control. For the PT assay, 50 μL of control or treated PPP was mixed with 100 μL of Thromboplastin-DS (Pacific Hemostasis, Thermo Fisher Scientific, Waltham, MA, USA) to initiate the extrinsic pathway cascade. For the aPTT assay, 50 μL of the PPP sample was pre-mixed with 50 μL of aPTT-XL reagent (Pacific Hemostasis, Thermo Fisher Scientific), and the intrinsic coagulation pathway was subsequently triggered by adding 50 μL of 25 mM CaCl_2_. The clotting times for both assays were recorded, with a detection interval spanning from 10 to 180 s [61,62]. Apixaban 0.2 μM was used as a control.

### 4.16. In Vitro Thrombosis Assay

In vitro thrombosis was evaluated under biomimetic shear conditions using a microfluidic perfusion system [63]. Briefly, citrated whole blood was labeled with the fluorescent dye DiOC6 (25 nM) for 1 h at 37 °C. Microfluidic channels (Vena8 Fluoro+ biochips) were coated with 2 μL of type I collagen (1 mg/mL) and incubated for 1 h at room temperature in a humidified chamber to promote platelet adhesion. Blood samples (700 μL) were pre-incubated with either peptide p23 (30 μM final concentration) or the vehicle control (distilled water). The biochips and perfusion tubing were assembled, and the treated or control blood was perfused through the collagen-coated channels at a shear stress of 20 dyn/cm^2^ for 10 min [64]. Following perfusion, the channels were gently washed with phosphate-buffered saline (PBS) to remove non-adherent blood cells. Thrombus formation and fluorescent platelet aggregate image acquisition were performed using a Nikon Ti2 confocal fluorescence microscope (Nikon, Tokyo, Japan) equipped with a 40× objective lens [65].

### 4.17. Analyses

The results obtained from the docking studies and molecular simulations were processed using custom Python v3.10 [66] scripts designed to extract binding energies, calculate key interatomic distances, and perform comparative analyses between the different peptides and the PDI catalytic sites. Additionally, conformational stability assessments were performed using parameters such as total energies, Root-Mean-Square Deviation (RMSD), and Root-Mean-Square Fluctuation (RMSF) of the simulated trajectories.

Experimental data from all in vitro and ex vivo biological assays (including cytotoxicity, PDI reductase activity, platelet aggregation, flow cytometry, microfluidic thrombosis, clot retraction, and coagulation parameters) are expressed as the mean ± standard deviation (SD). To ensure biological reproducibility, all functional assays were conducted utilizing independent experiments (*n* = 3 to 8 per assay), with blood-based assays utilizing samples from different healthy donors. Statistical analyses were performed using GraphPad Prism v9.0 (GraphPad Software, San Diego, CA, USA) [67]. Depending on the experimental design, significant differences between groups were determined using either a one-way analysis of variance (ANOVA) followed by Tukey’s post hoc test for single-factor multiple comparisons, or a two-way ANOVA followed by Bonferroni’s post hoc test for experiments evaluating multiple variables. A *p*-value < 0.05 was considered statistically significant.

## 5. Conclusions

This study validates the rational, bioinformatics-guided design of targeted peptides as a highly effective strategy for the development of novel antithrombotic agents. By exploiting the evolutionary conservation of the PDI catalytic site, we successfully engineered p23, a peptide that exerts its selective antiplatelet activity through a distinct covalent anchoring mechanism at Cys400, thereby inhibiting platelet aggregation and preventing thrombus growth dependent on platelet–platelet interactions. The ability of p23 to maintain consistent inhibitory efficacy in human plasma, selectively disrupt integrin activation, and prevent thrombosis under arterial flow without impairing essential hemostatic and coagulation processes underscores its immense translational potential. Consequently, peptide p23 emerges as a highly promising lead candidate for the management of atherothrombotic disorders, warranting further pharmacokinetic optimization and comprehensive evaluation in vivo animal models of thrombosis and hemostasis.

## Figures and Tables

**Table 1:** Docking results.

	Covalent Docking			Standard Docking		
Peptide	Score	Dihedral Angle	Distance S(Cys400)-S(Pep)Å	Energy (kcal/mol)	Key PDI Pocket Residues (Non-Covalent)	Distance S(Cys400)-S(Pep)Å
p7	−4.9	−143.1	Cys11 2.19	−8.8	Arg300, Asp2; Trp396, Ala5; Lys401, Phe3; Cys397, Trp7; Phe440, Trp7; His438, Trp7; Pro441,His10; Arg461, His10; Glu460, Lys12.	Cys8 10.79; Cys11 14.26
p21	−9	−168.7	Cys8 2.4	−6.0	Asp363, Lys12; Lys424, cys11; Lys424, His10; Gln402, Phe3; Arg461, Asp2.	Cys11 14.75; Cys8 14.30
p23	−7.49	−124.6	Cys11 2.20	−9.3	Asp363, Lys12; Asp426, Cys11; Pro395, Gly9; Trp396, Ala5; Cys397, Phe3; Gly398, Phe3; Phe440, Asp2.	Cys8 11.31; Cys11 12.63
p27	−6.12	−136.3	Cys8 2.11	−6.3	Phe440, Trp7; Phe304, Trp4; Arg300, Glu2; Ser439,Lys12; Lys436,Lys12.	Cys11 8.03; Cys8 12.98
p33	No poses obtained				No poses obtained	

**Table 2:** Inhibitory potency (IC_50_) of the candidate peptides on agonist-induced platelet aggregation.

	Washed Platelets			Platelet-Rich Plasma	
Peptide	Collagen (2 µg/mL)	Thrombin High-Dose (0.1 U/mL)	Thrombin Low-Dose (0.01 U/mL)	Collagen (2 µg/mL)	ADP (4 µM)
p7	42.6 ± 17.9 µM	>60 µM	>60 µM	>60 µM	>60 µM
p21	41.3 ± 16.5 µM	>60 µM	>60 µM	>60 µM	>60 µM
p23	34.1 ± 8.3 µM	>60 µM	>60 µM	53.9 ± 22.1 µM	>60 µM
p27	>60 µM	>60 µM	>60 µM	>60 µM	>60 µM
p33	>60 µM	>60 µM	>60 µM	>60 µM	>60 µM

## Data Availability

The raw data supporting the conclusions of this article will be made available by the authors on request.

## Supplementary Materials

The following supporting information can be downloaded at: https://www.mdpi.com/article/10.3390/molecules31193516/s1.

## Abbreviations

The following abbreviations are used in this manuscript:

ACD	Acid-citrate-dextrose
ACM	Acetamidomethyl
Ala/A	Alanine
ANOVA	Analysis of variance
AP	Average Precision
APTT/aPTT	Activated partial thromboplastin time
Arg/R	Arginine
Asp/D	Aspartic acid
AUC	Area Under the Curve
BSA	Bovine serum albumin
Cys/C	Cysteine
DTT	Dithiothreitol
EPT	Eptifibatide
ESM-2	Evolutionary Scale Modeling
FSC	Forward scatter
Gln/Q	Glutamine
Glu/E	Glutamic acid
Gly/G	Glycine
His/H	Histidine
HPLC	High-performance liquid chromatography
IC_50_	Half-maximal inhibitory concentration
Ile/I	Isoleucine
LDH	Lactate Dehydrogenase
Leu/L	Leucine
Lys/K	Lysine
MD	Molecular dynamics
MFI	Mean fluorescence intensity
MS	Mass spectrometry
NPT	Isothermal-isobaric
PBS	Phosphate-buffered saline
PDB	Protein Data Bank
PDI	Protein disulfide isomerase
Phe/F	Phenylalanine
PPP	Platelet-poor plasma
PR	Precision–Recall
Pro/P	Proline
PRP	Platelet-rich plasma
PT	Prothrombin Time
RMSD	Root-Mean-Square Deviation
RMSF	Root-Mean-Square Fluctuation
ROC	Receiver Operating Characteristic
SD	Standard deviation
Ser/S	Serine
SSC	Side scatter
Thr/T	Threonine
Trp/W	Tryptophan
Tyr/Y	Tyrosine
Val/V	Valine
