# Supplementary material for: Design and Evaluation of Novel Peptides Targeting Protein Disulfide Isomerase as Antiplatelet Agents
Source: Molecules. 2026 Oct 2;31(19):3516. doi: 10.3390/molecules31193516 (PMC13666787; doi:10.3390/molecules31193516)
Supplement: Supplementary file 1 [file molecules-31-03516-s001.zip › Rodriguez-Cabello F et al Molecules supporting information.pdf]

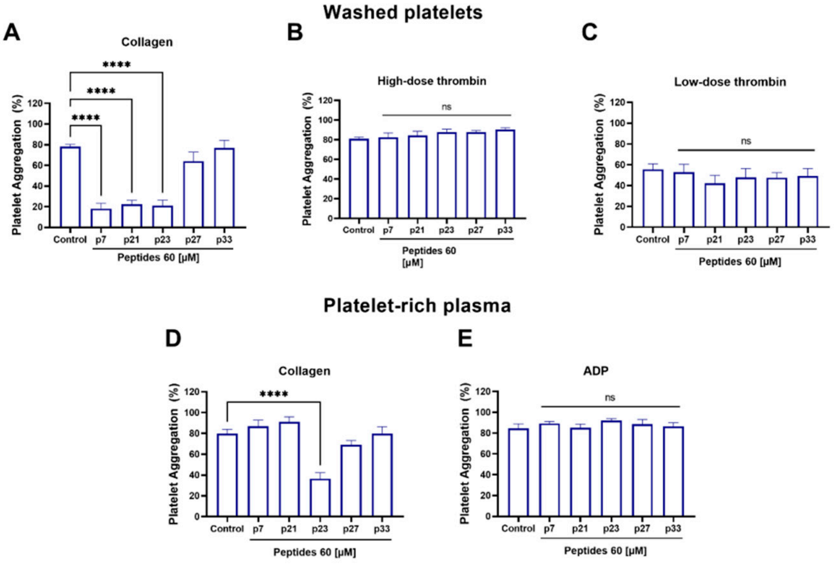

Supplementary Figure S1. Initial functional screening of candidate peptides on agonist-induced platelet aggregation at a fixed concentration. The inhibitory effect of the five candidate peptides (p7, p21, p23, p27, and p33) was evaluated at a single screening concentration of 60 μM. Platelet aggregation was assessed in washed platelets stimulated with (A) collagen (2 μg/mL), (B) high-dose thrombin (0.1 U/mL), and (C) low-dose thrombin (0.01 U/mL). Peptides p7, p21, and p23 demonstrated significant inhibition exclusively against collagen-induced aggregation in washed platelets. Additionally, aggregation was evaluated in platelet-rich plasma (PRP) stimulated with (D) collagen (2 μg/mL) and (E) ADP (4 μM). Only peptide p23 retained significant antiplatelet activity against collagen in the complex PRP environment. Thrombin- and ADP-induced aggregation remained unaffected across all tested peptides. Data are expressed as the percentage of maximum platelet aggregation (mean ± SD) of five independent experiments using blood from different healthy donors (n = 5). Statistical analysis was performed using one-way ANOVA followed by Tukey's post hoc test (ns: not significant, **** $p < 0.0001$ vs. uninhibited Control).

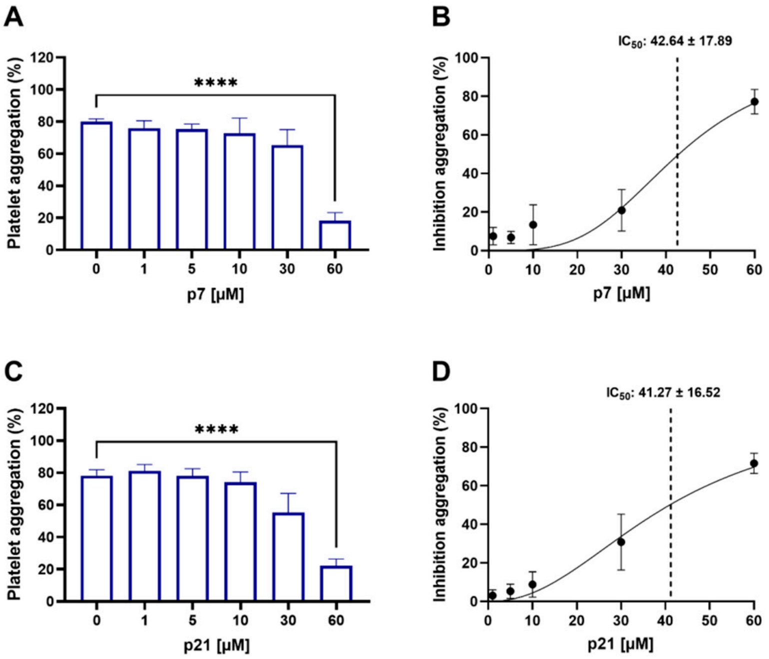

Supplementary Figure S2. Dose-response effect and IC50 determination of peptides p7and p21 on collagen-induced platelet aggregation. Following the initial screening, the dose-dependent inhibitory effects of peptides p7 and p21 were evaluated at concentrations ranging from 1 to 60 μM against collagen-induced aggregation (2 μg/mL) in washed platelets. (A) Bar graph displaying the absolute percentage of platelet aggregation in the presence of varying concentrations of peptide p7. (B) Non-linear regression curve used to calculate the half-maximal inhibitory concentration (IC50) for p7, plotted as the percentage of aggregation inhibition. The calculated IC50 is 42.64 ± 17.89 μM. (C) Bar graph displaying the absolute percentage of platelet aggregation in the presence of varying concentrations of peptide p21. (D) Non-linear regression curve used to calculate the IC50 for p21, with a calculated value of 41.27 ± 16.52 μM. Data are presented as the mean ± SD of five independent experiments using blood from different healthy donors (n = 5). Statistical significance was determined using one-way ANOVA (**** $p < 0.0001$ vs. 0 μM vehicle control).

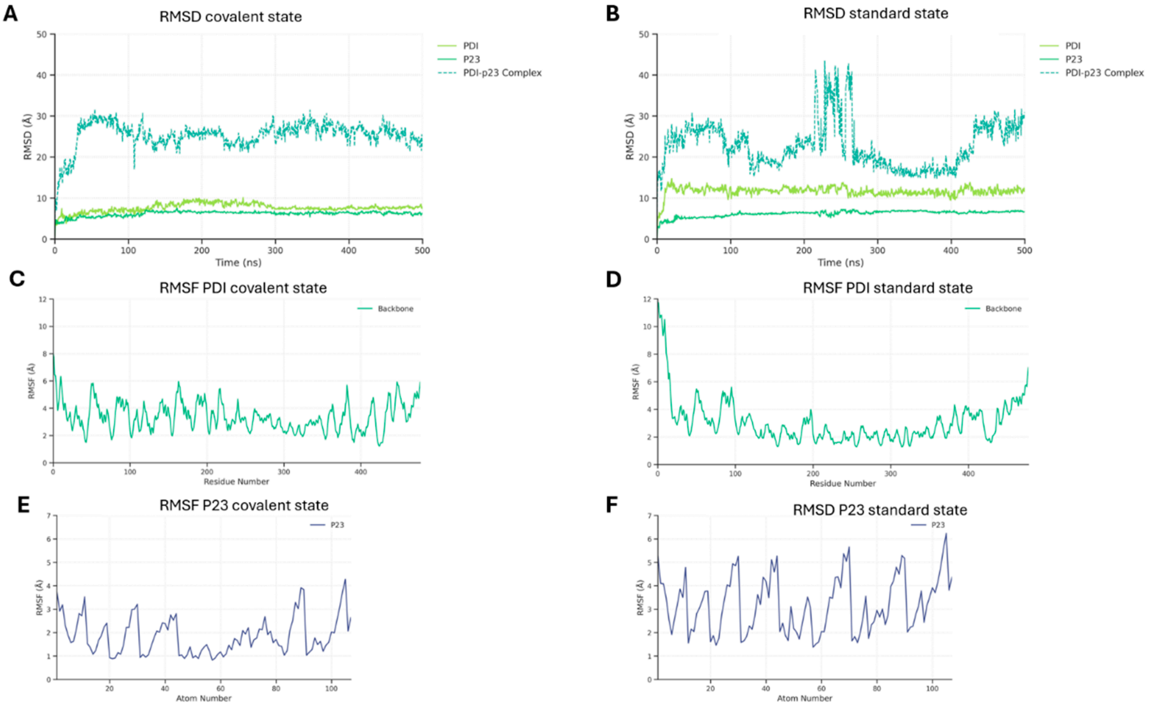

Supplementary Figure S3. Conformational dynamics of the PDI-p23 complex. Root-Mean-Square Deviation (RMSD) and Root-Mean-Square Fluctuation (RMSF) profiles over the 500 ns MD simulation. The left column displays the high structural stability of the covalent state, including (A) RMSD of the complex, (C) RMSF of the PDI backbone, and (E) RMSF of the p23 ligand. The right column shows the inherent flexibility of the standard non-covalent state, detailing (B) RMSD of the complex, (D) RMSF of the PDI backbone, and (F) RMSF of the p23 ligand.

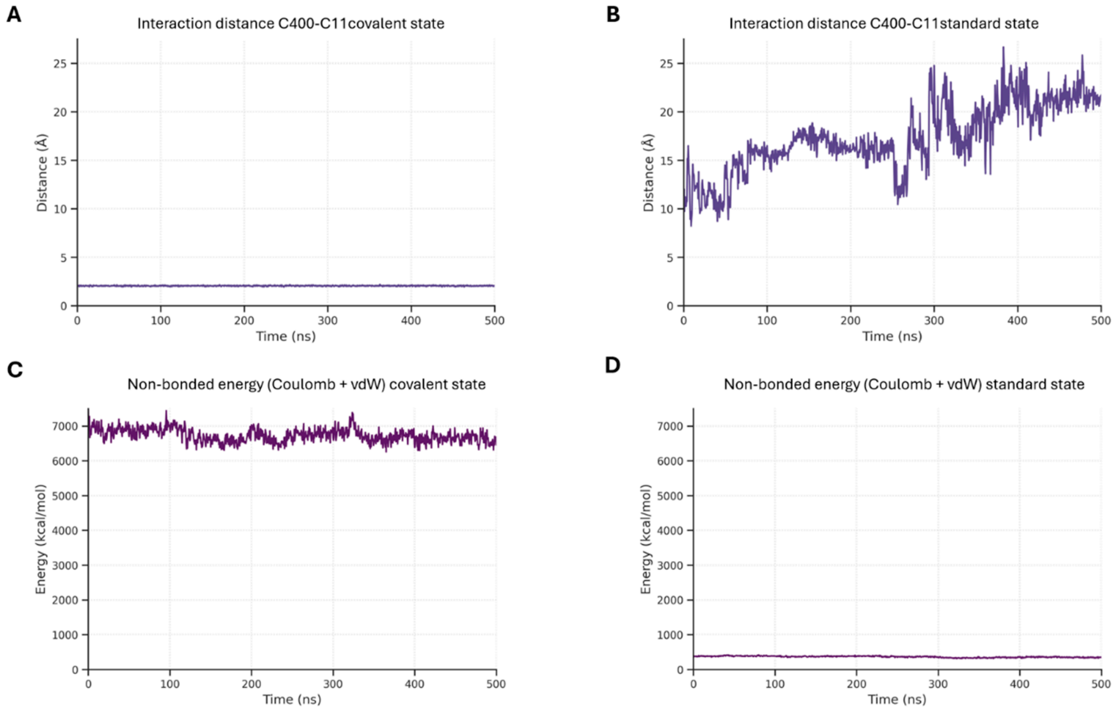

Supplementary Figure S4. Interaction distance and non-bonded energetic profiling. Analysis of key binding parameters during the 500 ns MD simulation. The inter-sulfur distance between PDI-C400 and p23-C11 is shown for (A) the covalent state, where it remains rigidly locked at ~2 Å, and (B) the standard state, where it dynamically fluctuates. Total non-bonded interaction energies (Coulomb + van der Waals) are plotted for (C) the covalent state and (D) the standard state, indicating consistent energetic stability over the simulated timeframe.

Supplementary Video S1. 500 ns molecular dynamics trajectory of the stable covalent PDI-p23 inhibitory complex.

Supplementary Video S2. 500 ns molecular dynamics trajectory of the dynamic non-covalent PDI-p23 encounter complex.

Supplementary Video S3. Real-time *in vitro* thrombus formation in untreated whole blood under arterial shear conditions. This time-lapse video demonstrates the perfusion of DiOC6-labeled citrated whole blood (vehicle control) through a type I collagen-coated microfluidic channel at a biomimetic arterial shear stress of 20 dyn/cm². Throughout the perfusion period, rapid platelet adhesion and robust aggregation are observed, culminating in the continuous formation and consolidation of large, stable fluorescent thrombi.

Supplementary Video S4. Real-time *in vitro* thrombus formation in whole blood pre-incubated with peptide p23. This time-lapse video demonstrates the perfusion of DiOC6-labeled citrated whole blood pre-treated with the lead candidate peptide p23 (30 μM) through a type I collagen-coated microfluidic channel under identical arterial shear stress conditions (20 dyn/cm²). In stark contrast to the vehicle control, the presence of p23 markedly impairs platelet aggregation and prevents the architectural development of stable three-dimensional thrombi over the course of the perfusion.

https://doi.org/10.3390/molecules31193516
